# Conservative Treatment of Dental Non-Carious Cervical Lesions: A Scoping Review

**DOI:** 10.3390/biomedicines11061530

**Published:** 2023-05-25

**Authors:** Assunta Patano, Giuseppina Malcangi, Matteo De Santis, Roberta Morolla, Vito Settanni, Fabio Piras, Alessio Danilo Inchingolo, Antonio Mancini, Francesco Inchingolo, Gianna Dipalma, Angelo Michele Inchingolo

**Affiliations:** Department of Interdisciplinary Medicine, University of Bari “Aldo Moro”, 70124 Bari, Italy; assuntapatano@gmail.com (A.P.); giuseppinamalcangi@libero.it (G.M.); m.desantis51@studenti.uniba.it (M.D.S.); robertamorolla@gmail.com (R.M.); v.settanni@libero.it (V.S.); dott.fabio.piras@gmail.com (F.P.); ad.inchingolo@libero.it (A.D.I.); dr.antonio.mancini@gmail.com (A.M.); giannadipalma@tiscali.it (G.D.)

**Keywords:** non-carious cervical lesions, resin-modified glass-ionomer cements, conservative, composite restoration, enamel abrasion, systematic review, RCT

## Abstract

This scoping review aims to evaluate methods of conservative reconstruction of dental enamel lesions resulting from abrasions and evaluate the effect of diode laser in reducing the symptoms of tooth sensitivity. The cementoenamel junction is more prone to substance loss because the enamel thickness is substantially decreased, resulting in a much weaker enamel–dentin bond. Methods: Dental abrasion was examined in the mechanical cause alone. Pubmed, Scopus, and Web of Science were used to discover publications that matched our topic from 1 January 2018 to 20 March 2023. A comparison of various non-carious cervical lesion (NCCL) restoration treatments was generated mostly by mechanical considerations. Results: A final number of 11 clinical trials and randomized controlled trials were included in the review for qualitative analysis. Composite resins performed well in clinical trials for the restoration of NCCLs. Conclusions: Composite, in its different forms of filling and consistency, preceded by the use of adhesives, is an efficient and effective material for the treatment of NCCLs. Diode laser use prior to NCCL restoration of teeth does not diminish restoration retention rate, may lessen hypersensitivity, and may affect restoration success.

## 1. Introduction

Non-carious cervical lesions (NCCLs) consist of irreversible loss of mineralized tissue unrelated to carious pathology [1,2,3]. Generally, NCCLs are located in the cervical third of the tooth at the level of the cementoenamel junction and tend to extend from the latter toward the tooth root [4]. The cementoenamel junction proves to be more prone to loss of substance because the thickness of the enamel is greatly reduced and, consequently, the enamel–dentin bond is much weaker [5].

Indeed, it has been seen that NCCLs are lesions present at all ages; however, epidemiological studies have pointed out a significant increase in their incidence at older ages [6,7]. Kolak et al. [8] found that in a group of patients older than 55 years, 94.7 percent of them had NCCLs, and one-third of them had more than three lesions.

The increase in life expectancy resulting in an older population requires some attention regarding certain oral health-related variables that may affect quality of life (QoL). Older age is related to an increase in chronic diseases but also dental and oral problems. Specifically, carious pathology and periodontal disease often worsen with advancing age resulting in a compromised masticatory system [9].

Generally, tooth hard-tissue loss could be classified as physiological or pathological. In the former case, tissue loss is a consequence of the chewing action over time and may also be localized to the interproximal level as a result of friction between the tooth elements [10,11].

On the other hand, a large loss of dental tissue is to be considered pathological as it causes progressive destruction of the tooth and may require intervention by a dentist [12,13,14].

**Figure 1:**
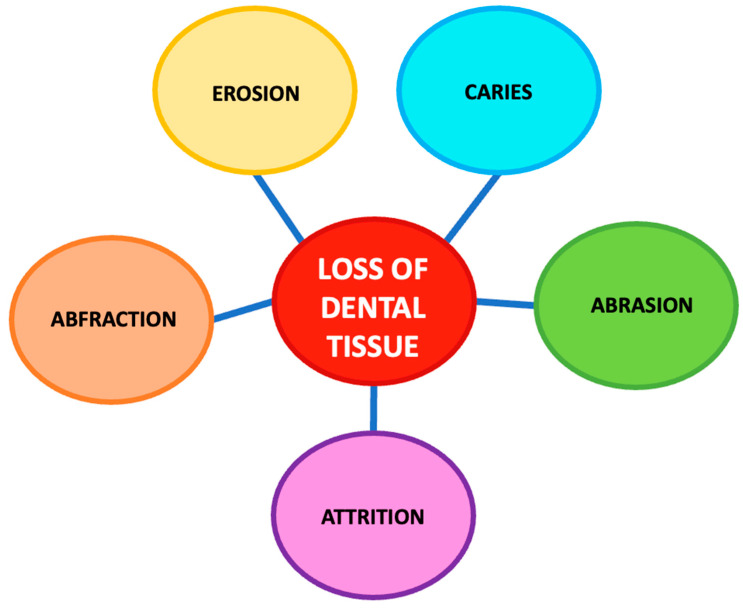
Diagram of the causes of tooth tissue loss.

NCCLs have a multifactorial etiology and are the consequence of several phenomena that may also occur simultaneously. Causes of NCCLs include abrasion, abfraction, erosion, and attrition [3,15,16] (Figure 1).

Correct identification of their cause allows not only to choose the most appropriate treatment plan, but also to reduce the progression of already formed lesions and place the patient on a prevention plan [17,18,19].

Specifically, abrasion and erosion are responsible for removing the smear layer or intratubular substance through a mechanical or chemical process, respectively [20].

The opening of dentinal tubules will be responsible for the increase of dentinal hypersensitivity at the level of NCCLs [21].

Two-step self-etch adhesives succeed in penetrating different types of smear layers due to the high hydrophilicity of the self-etching primer. In contrast, one-step self-etch adhesives have greater difficulty in penetrating the smear layer and difficulty in forming the hybrid layer with decreased adhesion strength to the dentin [22].

### 1.1. Dental Abrasion

Abrasion of the tooth surface is a consequence of external abrasive substances rubbing against it [23,24]. Abrasive factors include the use of a hard-bristled toothbrush associated with a vigorous brushing technique and abrasive toothpaste [25].

The distribution of lesions can help the dentist correctly identify risk factors. For example, unilateral localization of lesions at the level of the second quadrant may be associated with an improper brushing technique in a right-handed individual [26,27,28,29].

Abrasion lesions can also localize at the occlusal surfaces of the teeth as a result of an abrasive diet or at the chewing of abrasive materials such as tobacco [26].

### 1.2. Dental Abfraction

Abfraction consists of the pathological loss of tooth hard tissue as a result of compressive and tensile occlusal loading forces that cause microfractures at the level of the enamel and cervical dentin resulting in the weakening of the tooth structure [30,31,32,33].

Usually, abfraction lesions are located at the cervical level of the tooth on its buccal surface. They present in a wedge or V shape with well-defined margins [34].

This presentation of them makes them very similar to abrasion lesions and, therefore, they are difficult to distinguish from the latter because they are very often simultaneously present and tend to overlap [35].

### 1.3. Dental Erosion

Tooth surface erosion consists of the dissolution of mineralized tooth tissue as a result of the action of non-bacterial acids [23].

Factors affecting the severity of erosion include the amount and temperature of the acidic substance and the time the acid comes into contact with the hard tissue of the tooth.

The location of erosion lesions changes according to the origin of the acid substance. In this regard, erosion can be classified as exogenous or endogenous. In the case of endogenous erosion, the acid substance comes from the body itself such as gastroesophageal reflux. The latter will cause lesions on the palatal surfaces of the upper front teeth [36]. In the case of exogenous erosion, on the other hand, acidic substances are introduced from outside. In this case, acidic beverages or foods are the major culprits for the erosion of the buccal surfaces of the teeth [37]. Thus, as a result of the constant contact of the acidic substance with the tooth surface, there is a progressive softening of the dental hard tissue with subsequent dissolution and total loss [38].

### 1.4. Dental Attrition

Attrition wear is the physiological wear caused by opposing teeth contacting each other in the absence of an abrasive substance. The amount of applied load and the duration of load application both contribute to this form of wear [13,23,39]. Non-axial (lateral) loads associated with chronic clenching (parafunction) cause surface flexure in the cervical region that surpasses established enamel failure stresses [40,41].

### 1.5. Management of NCCLs

The level of severity of NCCLs results in various signs and symptoms that may include a progressive increase in dentinal hypersensitivity, impaired dental aesthetics, and subsequent plaque accumulation in the lesion area [15].

The loss of mineralized tissue resulting from the formation of NCCLs causes the formation of tertiary or reparative dentin, which obstructs the dentinal tubules [18,42,43].

The deposition of such reaction dentin can adversely affect the adhesion of restorative material to the tooth surface [23].

There are many other factors that can compromise the durability of the restoration, including the depth of the lesion, the shape of the cavity, and the restoration performed. Added to this is the position of the NCCL, and, if it turns out to be too cervical, there may be a risk of having an overly moist environment that is difficult to isolate [18,23,42,43].

For these reasons, it has been shown that the treatment and prediction of NCCLs can be difficult [44]. The ability of the chosen material to adhere to the tooth and resist occlusal forces determines the success of the restoration performed [45]. Treatment of NCCLs should be performed when it is an advanced and no longer superficial lesion in which topical hypersensitivity control has failed. It is imperative to intervene when it is difficult for the patient to maintain proper home oral hygiene, which may also be joined by an aesthetic demand on the part of the patient [46].

The purpose of this scoping review is to analyze conservative techniques for treating NCCLs and reducing their sensitivity. Contextually, not only were the restorative materials of NCCLs analyzed and compared, but also the various adhesive systems that can currently allow greater retention of the restoration over time.

## 2. Materials and Methods

### 2.1. Protocol and Registration

The current scoping review was carried out in compliance with the standards of the Preferred and the International Prospective Register of Systematic Review Registry guidelines (ID: 414639) [47].

### 2.2. Search Processing

Pubmed, Scopus, and Web of Science were searched to find papers that matched our topic dating from 1 January 2018 up to 20 March 2023. The search method was developed by combining phrases that suited the goal of our review, which deals with the comparison of various restorative techniques of NCCLs.

Hence, the following Boolean variable and keywords were used: “tooth surface loss” OR “tooth wear” OR “dental wear” OR “tooth abrasion” OR “tooth abfraction” OR “non-carious cervical lesions” AND (“therapy” OR “treatment”).

### 2.3. Inclusion Criteria

The articles were selected using the following inclusion criteria: (1) studies only on human subjects; (2) open access studies; (3) clinical trials and randomized controlled trials; (4) English language and (5) studies concerning the treatment through various techniques of NCCLs.

### 2.4. Data Processing

Two reviewers (R.M. and M.D.S.) searched the database to extrapolate the studies and assessed their quality independently, according to selection criteria. During the screening phase, we excluded articles that did not fit the topic by reading the manuscript title and the abstract. The full texts of the remaining articles were read to conduct an eligibility analysis, according to the inclusion criteria. The selected articles were downloaded in Zotero (version 6.0.15). Any discrepancies between the two authors were resolved by consulting a senior reviewer (F.I.).

### 2.5. PICOS Criteria

Table 1 depicts the PICOS (Population, Intervention, Comparison, Outcome, Study design) criteria components, which include population, intervention, comparison, outcomes, and research design, as well as their use in this evaluation.

## 3. Results

From the search of the records on the Scopus, PubMed, and Web of Science databases, 1006 articles were found, of which 492 duplicates of these records were excluded from the search before the screening.

**Figure 2:**
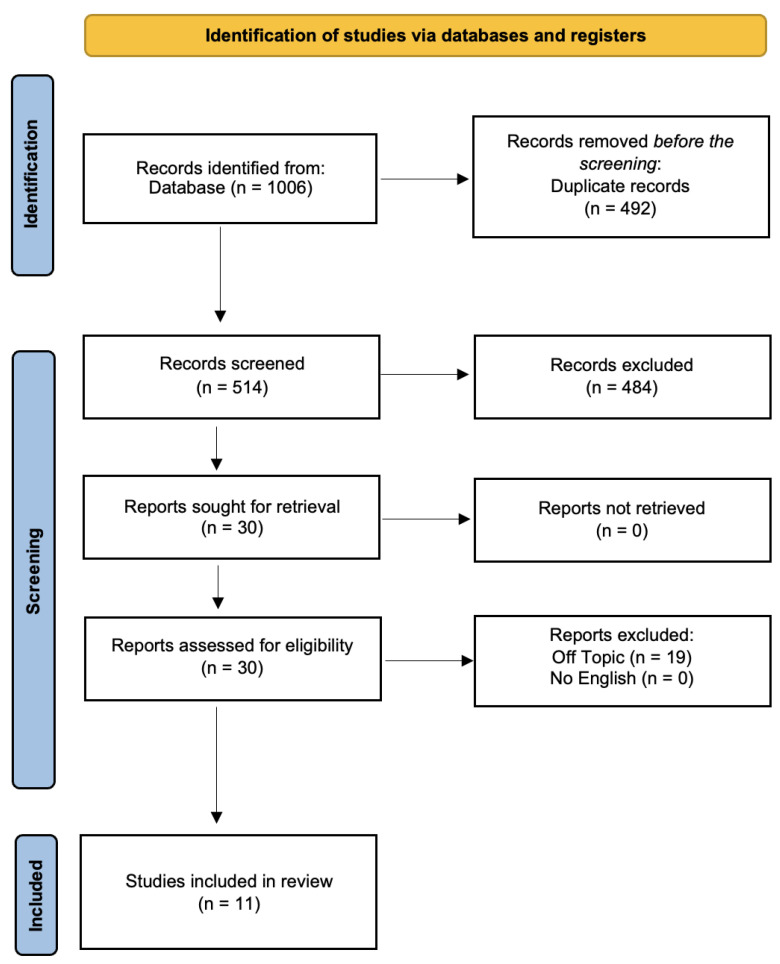
PRISMA flowchart diagram of the inclusion process. Literature search Preferred Reporting Items for Systematic Reviews and Meta-Analyses (PRISMA) flow diagram.

Hence, 514 items were reviewed, and 484 items were excluded because they were excluded from our Materials and Methods. A total of 30 reports were sought for retrieval and 0 reports were not retrieved. A total of 30 reports were assessed for eligibility and 19 reports were excluded. A final number of 11 studies were included in the review for qualitative analysis (Figure 2) (Table 2).

## 4. Discussion

It is well recognized that the etiology of NCCL is determined by a variety of variables, including the patient’s risk factor, which varies greatly, altering the therapeutic efficiency of restorative materials [48]. NCCLs are a common clinical manifestation with a complex etiology that increases with age. The absence of macro-mechanical retention and tiny C-factor in these class V cavities minimizes the effect of material features such as polymerization shrinkage, and hence restoration success is mostly dependent on the material’s real bonding capability [53]. The retention of NCCL restorations is closely connected to the restorative system’s adhesion capacity and elasticity modulus [56].

### 4.1. Analysis of Conservative Methods

In the current investigation, a significant relationship was discovered when clinical characteristics (retention, surface texture) of RMGIC/Flowable composite were compared. The resin-modified Glass Ionomer Cements (RMGIC) Group retained 93% of their restorations, inside the range of worldwide research. Nevertheless, only 63% of repairs in the Flowable Composite group were retained. The Flowable Composite Group had a marginal adaptation of 70%, whereas the RMGIC Group had a marginal adaptation of 76.6%. Flowable Composite Group was 60% and RMGIC Group was 83%. The Chi-square test revealed a significant connection between retention and both groups [48].

Based on the findings of this randomized controlled clinical trial’s 2 year follow-up, it is possible to conclude that GLUMA Universal and All-Bond Universal adhesives used in self-etch mode displayed inadequate retention rates at 6, 12, and 24 months. During the 2 years, there were negligible variations in marginal adaptation and marginal discoloration between universal adhesives in etch-and-rinse mode and Single Bond2. At the 24 month assessment, the etch-and-rinse and selective-etch application modes of GLUMA Universal and All-Bond Universal adhesives exhibited acceptable clinical results that were comparable to the evaluated etch-and-rinse adhesive system (Single Bond2) [50].

This is the first research to compare the clinical efficacy of a flowable bulk-fill composite to a standard nano-filled composite in the management of NCCLs. 

Their findings showed that both composites had acceptable clinical performances, despite minor changes in surface roughness, anatomic shape, and marginal adaptation after 1 year. In a recent study, a bulk-fill restorative composite outperformed a nano-filled normal composite in terms of marginal discoloration and marginal adaptation after 24 months in class II restorations.

The difference in elastic modulus between the two composites was ascribed to the findings, which may have produced greater contraction stresses and dislodgment of the nano-filled regular composite from the teeth borders, resulting in gap development and increased sensitivity to marginal staining [52].

Due to their excellent retention rates and simplicity of usage, GICs are the most popular substance. As compared to the baseline, the RMGIC group demonstrated dramatically improved but still tolerable marginal staining after 18 months. Their findings were consistent with previous research that found a rise in margin stain in RMGICs with time. Despite their good retention, RMGICs were shown to have higher water sorption and inferior aesthetic outcomes when compared to resin-based restorative materials. G-Bond (a mild one-step self-etch adhesive), on the other hand, exhibited no significant rise in marginal staining during an 18 month period. As it is HEMA-free, it is said to have less water sorption and hydrolytic deterioration over time. There was no postoperative sensitivity or secondary caries detected in any of the groups studied. This is thought to be due to the adhesives’ capacity to seal the dentinal tubules and minimize microleakage. In the repair of NCCLs, a moderate one-step self-etch adhesive followed by a resin composite restoration might be an alternative to RMGIC with comparable retention and enhanced aesthetics [53].

According to Luhrs et al. [54] the loss rate for restorations implanted without any dentin preparation was 7.7 years. Roughening the dentin surface and/or preparing a fine groove improved the long-term survival of restorations put in NCCLs and can be incorporated in the clinical treatment strategy for NCCLs. Due to the absence of mechanical retention, the clinical effectiveness of cervical restorations is mostly dependent on their adherence to the tooth structure. In this indication, one-step self-etch adhesives had a lower “clinical score” than two-step self-etch or three-step etch-and-rinse adhesives [54].

In their study, Luhrs et al. used the Syntac Classic adhesive in selective enamel etching mode (Ivoclar Vivadent, Schaan, Liechtenstein) in conjunction with the system-inherent self-etching primer. In NCCLs, this three-step adhesive (Syntac Classic) combined with selective enamel etching had the lowest yearly failure rate in a follow-up period of 13 years and is considered to be a reliable unfilled system [54].

Dentists offer a variety of restorative alternatives, with traditional RC being the most frequent, owing to its demonstrated lifespan and great aesthetics. Glass ionomers have also been advocated for this indication, owing to their less technically demanding use (e.g., moisture management) and the fact that these materials may be placed in a self-adhesive and bulk way. According to the study of Falk Schwendicke et al., in which they evaluated GH and RC for repairing NCCLs, the survival was not considerably different between GH and RC to repair sclerotic NCCLs. While GH was much less expensive both initially and over time than RC, utilizing RC was only cost-effective for payers prepared to incur higher expenditures for marginal survival increases [55].

The chemical interaction with the tooth structure is critical for the quality and stability of adhesion when healing these lesions. Due to their chemical interactions with the dentin substrates present in NCCL, glass-ionomer compounds improved retentive stability.

A comprehensive review and meta-analysis discovered that glass-ionomer restorations outperformed resin composite restorations in terms of retention [59]; however, other metrics revealed no significant difference. In their study, the group that did not achieve selective enamel conditioning had the highest initial debonding rates and the lowest 3 year cumulative survival (89%). The RMGIC group had a cumulative survival retention rate of 98%. Eighteen months following the operations, EDTA conditioning enhances self-etching adhesive repair retention rates. A dose of 0.1 M EDTA might be used as an alternate pretreatment for NCCL repair.

As compared to other pretreatment options for restorations using glass-ionomer cement, EDTA achieved reduced microleakage in primary teeth, constituting an alternative to improve chemical and micromechanical adherence of this material to the dental tissue [58].

At the end of the 24 month evaluation period, the whole clinical performance of the tested GH and nano-ceramic RBC restorative systems was equal for retention, marginal discoloration, secondary caries, postoperative sensitivity, and tooth vitality, with the nano-ceramic RBC system showing significantly better marginal adaptation.

Throughout the course of the investigation, a substantial shift in marginal discoloration was noted in both of the tested materials. Although the nano-ceramic RBC showed superior marginal adaptation than GH, both the evaluated GH restorative system and the present nano-ceramic RBC demonstrated a clinically acceptable performance for the restoration of NCCLs in bruxism patients [56].

Sensitivity is frequently found in NCCLs. Due to the obliteration of the dentin tubes by the adhesive and restoration, patients have reported diminished or resolved sensitivity following the restorative operation.

After 24 months of follow-up, just two teeth in each procedure (from the same individuals) still showed a degree of sensitivity as compared to the baseline condition, leading us to conclude that sensitivity may be inherited by patients. 

The semi-direct approach had no benefit over the standard direct method and was more time-demanding [49].

The efficacy of diode lasers on pain induced by DH in NCCLs was analyzed by comparing three alternative laser therapy protocols: high and low power were employed independently vs a hybrid technique employing first the low- and then the high-power laser. 

When the bio-stimulation impact of the delayed effect of the low-power laser on DH-associated reducing pain is combined with the quick pain control action of the high-power laser on external sealing, the optimal patient outcome is attained. 

They suggest an innovative laser therapy strategy that integrates two distinct diode laser output intensities to treat DH when its symptoms are triggered in the presence of NCCL to achieve more lasting pain control [57].

### 4.2. Effect of Diode Laser Application

Diode laser use prior to NCCL restoration of teeth does not diminish restoration retention rate, may lessen hypersensitivity, and may affect restoration success. This in vivo investigation found that groups who received a diode laser before restoration had poorer sensitivity than those that did not receive a laser before restoration [57].

## 5. Conclusions

Dental abrasions cause sensitivity to the patient; therefore, the ideal procedure is to treat them by reconstructing the lost tooth substance. The use of the diode laser as a desensitizing pre-treatment combined with the use of composite in its different forms of filling and consistency preceded by the use of adhesives make this material efficient and effective for the treatment of NCCLs. Therefore, composites are long-lasting materials for NCCL restoration. Further improvements in the properties of these materials will increasingly improve the loss rate of reconstructions.

## Figures and Tables

**Table 1:** PICOS Criteria.

Criteria	Application in the Present Study
Population	Subjects with NCCLs
Intervention	Conservative treatments of NCCLs
Comparisons	Comparing before and post intervention
Outcomes	Improvements as a result of the techniques used
Study design	Clinical Trial

**Table 2:** Descriptive summary of item selection.

Authors	Type of Study	Object	Study Design and Timeline	Type of Initial Restoration Placement or Repair of Existing Restorations	Lesions Sensitive or Not before Restoration	Conditioning Protocol Used for the Surface	Mechanical Preparation of the Surface	Results
Atikah Saghir et al. [48]	Randomized Clinical Trial	Comparison of the clinical efficacy of resin-modified glass ionomer and flowable composite in the treatment of NCCLs.	60 patients with at least 2 NCCLs divided into 2 groups: (1) treatment with flowable composite; (2) treatment with resin-modified glass ionomer cement.	Placement of new restoration in NCCLs with depth of 1–2 mm	Not specified	Tooth etching with 37% phosphoric acid	Not specified	NCCLs treated with Resin modified glass ionomer cement is superior to flowable composite in terms of retention and surface texture.
Taciana Marco Ferraz Caneppele et al. [49]	Randomized Clinical Study	To assess the effectiveness of resin composite restorations in NCCLs after 2 years utilizing direct or semi-direct techniques.	30 volunteers with at least 2 NCCLs. Each participant received one restoration with direct technique and one with indirect technique. Follow-up: baseline, 7 days, 6, 12 and 24 months.	New restorations have been made	Not specified	The surface was etched with 37% phosphoric acid gel	No tooth preparation was performed.	The tested restorative methods produce comparable effects for NCCLs during the study periods.
Fatma Dilsad OZ et al. [50]	Randomized Clinical Trial	To assess the effectiveness of two distinct universal adhesives and one etch-rinse glue for NCCL restoration.	20 patients with at least 7 NCCLs divided into 7 groups according to adhesive systems and application modes. Follow-up: 1 week, 6, 12 and 24 months.	New restorations of NCCLs have been made	Patients who rated their hypersensitivity with a maximum value of 7 on a scale of 0 to 10	Phosphoric acid etching gel (37%) was applied to enamel for 30s then rinsed and dried	Not specified	In terms of retention, GLUMA Universal and All- Bond Universal outperformed the self-etch mode in etch-and-rinse and selective etching modes.
Shanthana Reddy et al. [51]	Randomized Controlled trial	To assess the clinical efficacy of nanocomposite restorations for NCCLs bonded with universal adhesive in self-etch mode with and without air abrasive surface treatment.	70 NCCLs divided into 2 groups: (1) surface treatment with alumina air abrasion; (2) control group. NCCLs were restored with nanocomposite using universal bonding (self-etch mode). Follow-up: baseline, 3, 6, 12 months.	Teeth with cervical carious lesions and previously restored teeth.	Pre-operative sensistivity with Schiff’s scale: = (no response); 1 (mild response), 2 (moderate response); 3 (severe response)	Not specified	Lesion cleaned using pumice slurry	The clinical efficacy of nanocomposite resin bonded with universal adhesive was not improved by airborne particle abrasion of NCCLs.
Gabriela D. Canali et al. [52]	Randomized trial	To assess the efficacy of a bulk-fill flowable and a regular nanofilled composite in NCCLs after one year.	22 patients with at least 2 NCCLs. A universal adhesive was used with a self-etching approach in dentin. Follow-up: baseline (7 days), 6 months and 1 year.	New restorations of lesions	Not specified	32% phosphoric acid for 15 s on enamel	Enamel margins beveled with a diamond bur at high speed	After one year, both composite resins demonstrated acceptable clinical efficacy in the restoration of NCCLs.
M. Jassal et al. [53]	Randomized Clinical Trial	To compare the clinical efficacy of two techniques of applying a moderate one-step self-etch adhesive and composite resin in NCCLs with a resin-modified glass ionomer cement control repair (RMGIC).	294 restorations in 56 patients divided into 3 groups: (1) G-Bond active application combined with Solare-X composite resin; (2) G-Bond passive application combined with Solare-X composite resin; (3) GC II LC RMGIC. Follow-up: 6, 12 and 18 months.	New restorations of lesions ≥ 1 mm	18 of 294 NCCLs had preoperative sensitivity	Dentin conditioner for 10 s before RMGIC	Lesions were cleaned using rubber cup with pumice. No additional bevel	In the repair of NCCLs, a mild one-step self-etch adhesive followed by a resin composite restoration might be an alternative to RMGIC with comparable retention and enhanced aesthetics.
Anne-Katrin Lührs et al. [54]	Randomized Clinical Trial	To assess the clinical performance of restorations placed in NCCLs using different cavity preparation strategies.	85 NCCLs were treated with different cavity preparations and subsequent restoration.	New restorations of NCCLs have been made	Not specified	Selective enamel etching for 30 s with 36% phosphoric acid gel	In group 2 and 3 dentin surface is bevelled with round bur	Composites are long-lasting materials for NCCL restoration. Restorations that were put without any dentin preparation (just cavity cleaning) had the greatest loss rate.
Falk Schwendicke et al. [55]	Randomized Clinical Trial	To evaluate the survivability, restoration quality, and costs of glass hybrid and resin restorations on sclerotic NCCLs.	88 patients with 175 NCCLs were were randomized to receive glass hybrid (GH) or composite restorations (RC).	New restorations have been made	Not specified	For RC etching with 37% phosphoric acid gel	Surfaces cleaned using a polishing brush. No mechanical preparation	There was no significant difference between GH and RC in the survival and quality of NCCLs restorations. In addition, GH was found to be less expensive than RC.
Uzay Koc Vural et al. [56]	Randomized Clinical Trial	To compare the clinical efficacy of a glass hybrid restorative to that of a nano-ceramic composite resin in non-carious cervical lesions (NCCLs) of bruxism patients.	A total of 148 NCCLs were restored in a random order using either a glass hybrid (GH) restorative system or a nano-ceramic composite resin (RBC). Follow-up: at baseline, six, twelve, and twenty-four months later.	New restorations	61 of 148 NCCLs had preoperative sensitivity	RBC restorations was etched for 15 s with 37% phosphoric acid gel	Not specified	After 24 months both evaluated restoratives demonstrated clinically acceptable performance for the restoration of NCCLs in bruxism patients.
S Akarsu et al. [57]	Clinical Trial	To assess the impact of the diode laser used for DH on the clinical success of NCCLs repaired with various adhesive solutions.	20 NCCLs were restored with Universal Single Bond and Grandio after diode laser application, and 20 NCCLs were restored with Universal Single Bond (Total Etch) and Grandio. Follow-up: baseline, 6, and 18 months.	New restorations	NCCLs with tolarable sensitivity	Group 1: self-etch; Group 2: etch-and-rinse; Group 3: diode laser + self-etch; Group 4: diode laser + etch-and-rinse	No preparation on dentin or enamel.	Diode laser use prior to NCCL restoration of teeth does not reduce restoration retention rate, may decrease DH, and may affect restoration success.
Diego Felipe Mardegan GONÇALVES et al. [58]	Randomized Clinical Trial	To assess NCCLs restored with different adhesion techniques.	200 NCCLs were restored with Universal Single Bond with and without enamel conditioning; resin-modified glass-ionomer cements with EDTA. Follow-up: baseline and 3 years after.	New restorations	Visual analogue scale (VAS) was used to score pain: 1 (no pain); 2 (mild); 3 (moderate); 4 (slightly worse); 5 (much worse); 6 (severe pain)	Single Bond Universal was assessed both with or without enamel conditioning	No cavity preparation	NCCL restoration retention was impacted by selective enamel etching. The EDTA-based adhesive approach followed by RMGIC postponed marginal flaws over time.

## Data Availability

Not applicable.

## Abbreviations

DH	dentin hypersensitivity
EC	enamel conditioning
EDTA	ethylenediaminetetraacetic acid pretreatment
GH	glass hybrid
NCCL	non-carious cervical lesion
NCCLs	non-carious cervical lesions
NR	New restoration
RBC	nano-ceramic composite resin
RC	composite restoration
RMGIC	glass ionomer cement control repair
RMGIC	Resin-modified glass-ionomer cements
SBU	Single bond universal/Filtek Z350xt
